# Neck-specific strengthening exercise compared with placebo sham ultrasound in patients with migraine: a randomized controlled trial

**DOI:** 10.1186/s12883-022-02650-0

**Published:** 2022-04-02

**Authors:** Mariana Tedeschi Benatto, Lidiane Lima Florencio, Marcela Mendes Bragatto, Fabíola Dach, César Fernández-de-las-Peñas, Débora Bevilaqua-Grossi

**Affiliations:** 1 Department of Health Sciences, Ribeirão Preto Medical School, University of São Paulo, Ribeirão Preto, SP Brazil; 2 Department of Physical Therapy, Occupational Therapy, Rehabilitation and Physical Medicine, Universidad Rey Juan Carlos, Alcorcón, Spain; 3 Department of Neurosciences and Behavioral Sciences, Ribeirão Preto Medical School, University of São Paulo, Ribeirão Preto, SP Brazil

**Keywords:** Migraine disorders, Neck pain, Craniocervical exercises, Electromyography

## Abstract

**Background:**

Migraine patients have musculoskeletal disorders and pain in the cervical. And, despite the pathophysiology demonstrating the relationship between migraine and the cervical spine, the effectiveness of craniocervical exercises in these patients has not been verified. So, the aimed of this study was verify the effectiveness of craniocervical muscle-strengthening exercise (CMSE) in reducing the frequency and intensity of headache in migraine patients.

**Methods:**

A two-armed, parallel-group randomized controlled trial with a 3-month follow-up was performed. For eight weeks, the volunteers in the intervention group (*n* = 21) performed a protocol of CMSE, while those in the sham ultrasound group (*n* = 21) received the application of disconnected therapeutic ultrasound in the upper trapezius and guideline for home-stretching. The primary outcomes were the frequency and intensity of the headache. The secondary outcomes were questionnaires about migraine and neck disability, and satisfaction with the treatment, cervical range of motion, the pressure pain threshold, craniocervical flexion test (CCFT), cervical muscle strength and endurance test, and the cervical muscle activity during the physical tests.

**Results:**

No differences were observed for the changes observed in primary outcomes after eight weeks and at the 3-months follow up (*p* > 0.05). For the secondary outcomes, craniocervical exercises improved the sensitivity of the frontal muscle (*p* = 0.040) and promoted a reduced amplitude of muscle activity of the anterior scalene and upper trapezius in the last stages of CCFT (*p* ≤ 0.010). There was also reduced muscle activity of the anterior scalene and splenius capitis in the endurance test (*p* ≤ 0.045), as evaluated by surface electromyography.

**Conclusion:**

CMSE were insufficient in reducing the frequency and intensity of headache, improving the performance of the cervical muscles, or reducing migraine and neck pain-related disabilities. This was found despite a decreased electromyographic activity of the cervical muscles during the last stages of CCFT and increased median frequency during the endurance test.

**Trial registration:**

Accession code RBR-8gfv5j, registered 28/11/2016 in the Registro Brasileiro de Ensaios Clínicos (ReBEC).

**Supplementary Information:**

The online version contains supplementary material available at 10.1186/s12883-022-02650-0.

## Background

Patients with migraine may experience neck pain [1], worsened performance of cervical muscles [2–4], reduced cervical spine range of motion [5, 6], and increased muscle sensitivity in the craniocervical region [7, 8]. It can be explained by the pathophysiology of the disease since painful afferences from the upper cervical nerves and the trigeminal nerve converge at the trigeminocervical complex [9, 10].

Thus, currently, the management of migraine can include pharmacological treatment, considered the gold standard, and non-pharmacological therapies that can be used as an adjunct therapy to pharmacological treatment [11]. Physiotherapy is an important non-pharmacological treatment option for reducing the duration and frequency of migraine attacks and may include manual therapy, soft-tissue techniques, and strength and endurance training [12].

Exercises are also indicated for managing headaches and can be divided as: aerobic exercise or localized muscle-strengthening exercise [13]. Aerobic exercises reduce the frequency of migraine attacks and can be a prophylactic treatment option [14, 15]. Localized exercises, which include craniocervical muscle-strengthening exercise (CMSE), are widely used in the treatment of tension-type headaches and cervicogenic headaches [16, 17]. According to the current literature, strength exercises have a moderate clinical effect only in patients with tension-type headache. However, with a low quality of evidence [18]. Although pathophysiology has demonstrated a relationship between migraine and cervical spine in migraine patients [9, 10], there is no evidence on the effectiveness of localized exercises, including CMSE.

The choice in relation to CMSE was due to some aspects. Patients with migraine present a high prevalence of neck pain [19] and may also present dysfunctions in the cervical region, such as greater sensitivity of the craniocervical muscles, reduced force production of the cervical extensor muscles and worse performance of the deep flexor muscle, reduced ROM of cervical spine, hypomobility of the upper cervical segments (C1-C2) [6, 20]. In addition, migraine patients present many characteristics of central sensitization during the ictal and interictal phases. Central sensitization is also seen in patients with chronic neck pain and is an important characteristic of nociplastic pain. That is, patients with migraine, in addition to having a high prevalence of neck pain, have the same pain mechanism observed in patients with chronic neck pain. Update suggestions for the treatment of patients with mechanisms of nociplastic pain are centered in top-down strategies, such as exercises [21, 22].

The aim was to verify the effectiveness of CMSE in reducing the frequency and intensity of headache in migraine patients. We hypothesize that craniocervical exercise will reduce the frequency and intensity of headache. In addition, the craniocervical exercise intervention protocol may improve the performance of the cervical muscles, increase cervical range of motion, and increase the sensitivity of the craniocervical muscles. It is also expected to reduce migraine and neck pain-related disabilities.

## Methods

### Aim and design

This two-armed, parallel-group randomized controlled trial was designed according to the CONSORT extension for nonpharmacologic guidelines [23] to verify the effectiveness of craniocervical exercises in migraine patients. The protocol study was published [24]. The trial was approved by the Research Ethics Committee of the Hospital das Clínicas, Faculty of Medicine of Ribeirão Preto, University of São Paulo (no. 6862/2016).and registered under the accession code RBR-8gfv5j on 28/11/2016 in the www.ensaiosclinicos.gov.br/rg/RBR-8gfv5j/. All participants provided written informed consent.

### Participants and setting

Men and women aged between 18 and 55 years, diagnosed only with migraine and at least three days of pain per month were included. The diagnosis of migraine was confirmed by a neurologist with over five years of experience, per the 3^rd^ edition of the International Headache Classification [25]. The volunteers were recruited from the local population after the study was released on social media (Facebook®, Instagram®, and the local university radio). The exclusion criteria comprised of: the presence of other concomitant headaches, including headache due to medication abuse, tension-type headache or trigeminal autonomic cephalalgias; a history of trauma to the neck or face; a history of a herniated disc or joint degeneration in the cervical region; and systemic diseases such as fibromyalgia and uncontrolled arterial hypertension; pregnancy; local anesthetic nerve block in the last three months and having performed physical therapy in the craniocervical region in the last year. The data collection and the interventions were carried out in the laboratory of the research group.

Included participants underwent baseline assessment, which evaluated demographic characteristics, primary and secondary outcomes. Then, the participants were randomly assigned to the two groups and the interventions started in both groups in up to seven days. After six weeks of treatment, an intermediate assessment of CCFT and the Global Perception of Change questionnaire was done. After eight weeks of treatment, the final assessment was carried out and all the questionnaires and physical tests were applied. Follow-up via telephone was performed one, two, and three months after the end of the treatment only for the primary outcomes [24]. All participants were advised to do not start any type of physical therapy during the study duration, including the follow-up period and, due to ethical issues, they were advised to maintain their pharmacological treatment as they are used to before the inclusion in our study.

### Interventions

The single therapist responsible for both interventions, CMSE and sham ultrasound plus home-stretching, was trained to maintain standardization and adherence to the proposed interventions protocols. The therapist training so that the proposed intervention protocols were strictly followed is a measure to avoid the occurrence of bias [26].

The intervention group performed an eight-week CMSE protocol consisting of specific exercises for cervical muscles [24, 27]. The first stage, which lasted for six weeks, focuses on the deep cervical muscles [27]. It was proposed to start with two sets of 10 repetitions with 10 s of endurance for deep flexor muscles of the cervical and two sets of 10 repetitions for the deep extensor muscles. Progression in the number of series, repetitions, and endurance during the six weeks occurred individually, according to the complaint or absence of pain and execution of the movement without compensation, such as retraction or elevation of the head and excessive use of superficial flexors. The second stage lasted for two weeks, and in addition to the deep cervical muscles, it involved the superficial muscles of the cervical region [27]. It consisted of three sets of 15 repetitions for both the flexor and cervical extensor muscles. All volunteers received instructions from a physiotherapist, who had five years of experience, in individual sessions once a week for approximately 20 min. In addition, the volunteers were instructed to perform the craniocervical exercises at home twice daily and cervical muscle stretches. During the weekly sessions with the therapist, home guidelines were reinforced but the volunteers’ adherence was not monitored.

The volunteers in the sham ultrasound group had individual sessions once a week for approximately 20 min, in which disconnected therapeutic ultrasound was performed bilaterally in the middle portion of the upper trapezius for eight weeks. Volunteers also received a guideline book containing only cervical muscle stretching exercises to be performed once daily (Additional file 1) Stretching exercises for the cervical flexors, extensors, tilters and rotators were oriented. For this group, home guidelines on cervical muscle stretches were not reinforced weekly during the sessions and the performance was not monitored”.

If any volunteers from either group interrupted the treatment and left the study, contact was made, and continuity was requested only in the assessments.

### Outcomes

The primary outcomes were frequency (days with headaches per month) and intensity of headache by the numerical rating scale (NRS; 0–10) [28], collected using a paper headache diary. The gold standard for measuring data on the frequency and duration of headaches is self-report [29], and the use of headache diaries in randomized clinical trials is an important option to more accurately capture the effects of the interventions applied. in these outcomes [30]. The secondary outcomes were migraine and neck disability, collected using the Migraine Disability Assessment (MIDAS) [31, 32] and the Neck Disability Index (NDI) [33, 34], respectively; presence and severity of cutaneous allodynia verified by the 12-item Allodynia Symptom Checklist (ASC-12) [35]; presence of kinesiophobia detected by the questionnaire Tampa Scale for Kinesiophobia (TAMPA) [36]; functional changes over time related to migraine verified by the Patient-Specific Functional Scale (PSFS) [37]; and perceptions of improvement and satisfaction associated with an intervention, verified by the Global Perception of Change questionnaire [38]. It further included cervical range of motion, pressure pain thresholds, and performance of cervical muscles in CCFT, maximal isometric voluntary contraction (MIVC), and endurance with electromyographic records.

Cervical range of motion for flexion, extension, lateral flexion, and rotation were performed using the Cervical Range of Motion (CROM) (Performance Attainment Associates, Roseville, MN, USA) [39]. Pressure pain thresholds were assessed using a digital manual dynamometer (DDK-10 Kratos®) on the upper trapezius [7], sternocleidomastoid (SCM) [40], suboccipital, temporal [41] frontal [42] and levator scapulae muscles.

During the CCFT, MIVC, and endurance tests, muscle activity was recorded using surface electromyography. Wireless surface sensors (Trigno™ Wireless Systems, Delsys, Inc. Boston, MA, USA) were fixed with double-sided adhesive tape bilaterally over the SCM [43], anterior scalene [43], splenius capitis [44], and upper trapezius [45]. Data were processed by a customized MATLAB® routine employed to filter them at a frequency range of 20–450 Hz (4th order Butterworth), using a 0.375 s movable window with 50% overlap. The root mean square (RMS) from the CCFT records were normalized by their respective peak values during MIVC. For MIVC, RMS the mean RMS of each muscle was normalized to its peak value within the task. For the cervical muscle endurance test, the variables analyzed were the RMS slope and median frequency. To obtain them, initially, three windows were separated from the total acquisition window, 10% initial, 10% intermediate, and 10% final, thus creating a straight line of three points.

In the CCFT, volunteers remained in the supine position; a Stabilizer Pressure Biofeedback® device (Chatanooga, Hixson, TN, USA) was positioned at the back of the neck and, for each of the five stages, they were instructed to maintain the established pressure for 10 s without compensating for retraction or elevation of the head and excessive use of superficial flexors [46].

Muscle strength was measured. by a hand-held dynamometer (Lafayette Instrument Company®, model 2,201,163, Lafayette, IN, USA). To measure the strength of the cervical flexor muscles, the volunteers remained in the supine position, while for the extensor muscles, the volunteers remained in the prone position [47].

For the cervical flexor endurance test, the volunteers remained in the supine position and performed head and neck flexion without leaning their heads against the stretcher. For the cervical extensors, the volunteers remained in the prone position; the task was to keep the cervical muscles in a neutral position controlled by CROM and support a weight of 2 kg. The endurance test was interrupted in case of pain or fatigue, or inability to maintain the position [4]. Endurance time was measured in seconds and one repetition was performed.

### Sample size

The sample calculation was based on a between-group difference of 3.3 (standard deviation, SD = 3.1) days of headache per month [48] and 1.5 (SD = 1.4) in the NRS [49] considering a level of significance (α) of 0.025, a power of 80%, and a loss of 20% at follow-up. A sample size of 21 individuals from each group was recommended.

### Randomization

All volunteers were allocated in sequential order, determined by randomization using Excel®. The brown and opaque envelopes containing the group assignments were opened in front of each volunteer. A single therapist, blinded to the results obtained during evaluations, was responsible for all interventions in both groups. Similarly, the therapist responsible for data collection was blinded to the treatment allocation group. Both therapists were not blinded to the diagnoses because all the volunteers had migraine.

### Statistical analyses

Mixed linear regression models were performed for the primary and secondary results of the quantitative variables to estimate comparisons between groups and within the group. “Time” and “group” were considered as fixed effects and the participants as a random effect (random interception model). The presence and intensity of neck pain was used as a covariate, based on the difference observed between the groups at baseline. For comparison, a post-test using orthogonal contrasts was used. A multinomial regression model with mixed effects was used for comparisons involving the global change perception questionnaire. The analyses were performed with the aid of SAS 9.4 and R 3.5.3. A power of 80% was considered, and a significance level (α) of 0.05 was adopted for all comparisons.

The minimum important difference (MID) and effect size (ES) were calculated based on the distribution methods to attribute clinical relevance to the differences found in both groups. The difference found was considered clinically relevant when it was greater than the MID and was combined with an ES > 0.4 [50].

## Results

**Fig. 1:**
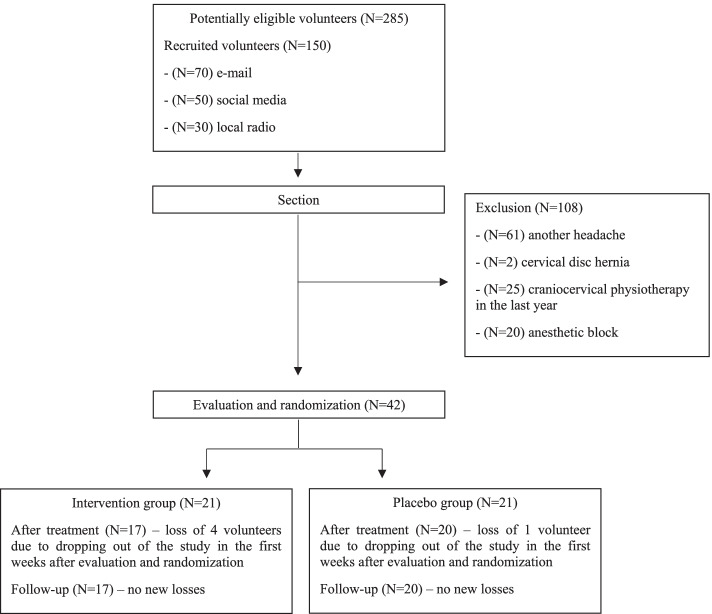
Recruitment and randomization of study patients

Forty (95.2%) women and two (4.8%) men were evaluated and randomized between June 2019 and February 2020. Of these, 5 (11.9%) volunteers did not continue the study for personal reasons (Fig. 1). The groups only differed in the baseline prevalence of self-reported neck pain and intensity, which was more prevalent and severe in the intervention group (*p* = 0.046 and *p* = 0.004, respectively), which is the reason why we considered both as covariate for the further analysis (Table 1).

**Table 1:** Baseline data

		**Intervention** **group (*N* = 21)**	**Sham ultrasound** **group (*N* = 21)**	***p* value**
*Fixed variables*				
Age (years)^a^		32.81 (8.59)	32.52 (8.91)	0.914
BMI (kg/cm^2^)^a^		24.52 (4.01)	24.96 (4.23)	0.645
Years of illness		15.33 (6.81)	15.38 (9.26)	0.747
Self-report of neck pain^b^		18 (85.71%)	10 (47.62%)	0.046*
Intensity of neck pain (NRS) ^a^		4.23 (2.64)	1.95 (2.33)	0.005*
Prophylactic medication^b^		8 (38.10%)	2 (9.52%)	0.144
Acute medication^b^		10 (47.62%)	12 (57.14%)	0.879
*Primary outcomes*				
Frequency of headache (days with headache/month)^a^		9.81 (8.80)	9.57 (6.88)	0.916
Intensity of headache (NRS)^a^		8.67 (1.11)	8.76 (1.51)	0.866
*Secondary outcomes*				
Cervical ROM^a^	*Movement*			
	Flexion	62.48 (7.99)	60.24 (9.83)	0.394
	Extension	73.86 (13.36)	72.57 (11.04)	0.727
	Lateral flexion right	46.05 (5.51)	48.05 (8.26)	0.403
	Lateral flexion left	51.86 (8.24)	48.52 (8.90)	0.214
	Rotation right	68.19 (7.78)	66.24 (5.36)	0.373
	Rotation left	69.29 (9.26)	67.62 (6.94)	0.507
Pressure pain threshold^a^	*Muscle*			
	Frontal	2.05 (0.72)	2.45 (1.02)	0.094
	Anterior temporalis	1.76 (0.62)	2.02 (0.83)	0.267
	Medium temporalis	2.20 (0.84)	2.39 (0.97)	0.498
	Posterior temporalis	2.30 (0.91)	2.58 (1.07)	0.359
	Sternocleidomastoid	1.85 (0.68)	2.21 (0.80)	0.122
	Scapular levator	2.45 (1.02)	2.78 (1.17)	0.315
	Upper trapezius	2.05 (0.72)	2.32 (0.91)	0.328
	Suboccipital	1.81 (0.64)	2.15 (0.76)	0.106
CCFT (mmHg)^a^	*Pressure level*			
		25.14 (1.96)	24.95 (2.58)	0.794
MIVC^a^	*Muscle group*			
	Flexor			
	Force_n_ (N/kg)	0.78 (0.29)	0.87 (0.20)	0.227
	Peak time (s)	2.30 (0.54)	2.31 (0.55)	0.927
	Extensor			
	Force_n_ (N/kg)	1.12 (0.50)	1.23 (0.49)	0.456
	Peak time (s)	2.50 (0.46)	2.65 (0.38)	0.257
Endurance^a^	*Muscle group*			
	Flexor endurance time (s)	44.24 (40.28)	39.90 (25.99)	0.651
	Extensor endurance time (s)	174.43 (128.25)	229.19 (170.87)	0.238
Questionnaires^a^	*Total score*			
	MIDAS	58.38 (46.25)	45.05 (27.00)	0.199
	NDI	13.00 (7.42)	9.38 (4.29)	0.072
	ASC-12	9.76 (4.11)	7.76 (3.63)	0.118
	TAMPA	37.48 (11.25)	35.1 (6.66)	0.377
	PSFS	4.32 (2.52)	4.94 (2.55)	0.190

*BMI* body mass index, *NRS* numerical rating scale, *ROM* range of motion; *mmHg* millimeters of mercury, *CCFT* craniocervical flexion test, *MIVC* maximal isometric voluntary contraction, *Force*_*n*_ force normalized by the subject mass, *s* seconds, *MIDAS* Migraine Disability Assessment, *NDI* Neck Disability Index, *ASC-12* 12 item Allodynia Symptom Checklist, *TAMPA* Tampa Scale for Kinesiophobia, *PSFS* Patient-specific Functional Scale

^a^data represented in mean and standard deviation

^b^data represented in N and percentage

^*^p < 0.05

### Primary outcomes

Frequency of headache: There were no statistically significant differences in the group-by-time interaction for the frequency of headache (F = 0.44, df = 4; *p* = 0.782). There were also no within-group differences in headache frequency during the study period (*p* > 0.05) (Table 2) as well as clinically relevant differences. All data used to classify the clinical relevance of the results related to the primary outcomes can be found at Additional File 1. (Table 2 and Additional file 2).

**Table 2:** Comparation within-groups and differences group-by-time interaction for primary outcomes

		**Baseline** **Final assessment**		**Baseline** **Follow-up 1**		**Baseline** **Follow-up 2**		**Baseline** **Follow-up 3**		Interaction group*time
		*Mean change (95%CI)*	*p value*	*Mean change (95%CI)*	*p value*	*Mean change (95%CI)*	*p value*	*Mean change (95%CI)*	*p value*	*F; df; p value*
*Primary outcomes*	*Groups*									
Frequency of headache^a^	IG SUG	1.13 (-2.29; 4.57) 1.16 (-2.06; 4.38)	0.513 0.477	1.19 (-2.23; 4.62) 1.01 (-2.21; 4.23)	0.492 0.536	2.07 (-1.35; 5.51) 0.41 (-2.81; 3.63)	0.233 0.801	2.43 (-1.00; 5.86) 0.01 (-3.21; 3.23)	0.163 0.994	0.44; 4; 0.782
Intensity of pain (NRS)	IG SUG	2.96 (2.03; 3.90) 3.73 (2.85; 4.62)	0.001^*CR^ 0.001^*CR^	3.31 (2.38;4.25) 2.78 (1.90; 3.67)	0.001^*CR^ 0.001^*CR^	3.96 (3.03; 4.90) 3.53 (2.65; 4.42)	0.001^*CR^ 0.001^*CR^	4.02 (3.08;4.96) 3.38 (2.50; 4.27)	0.001^*CR^ 0.001^*CR^	1.51; 4; 0.202

*95%CI* 95% confidence interval, *IG* intervention group, *SUG* sham ultrasound group, *NRS* numerical rating scale

^a^represented in days with headache per month

^*^p < 0.005

^CR^clinically relevant

Intensity of headache: There were no statistically significant differences in the group-by-time interaction for pain intensity (F = 1.51; df = 4; *p* = 0.202), although both groups showed significantly reduced intensity of headache in all comparison periods (*p* < 0.05) (Table 2). Clinically relevant within-group results were observed at some time points in the study for the intensity of headache. These results are described in Table 2 and Additional file 2.

### Secondary outcomes

Cervical range of motion: There were no significant differences in group-by-time interaction for any of the movements assessed during the study period (*p* > 0.05) (Table 3). Clinically relevant results were observed for some movements in both groups; these results are described in Table 3 and Additional file 3, in which all data used to classify the clinical relevance of the results related to the secondary outcomes can be found.

**Table 3:** Mean change and 95% confidence interval and differences group-by-time interaction for secondary outcomes and questionnaires

		**Baseline – Final assessment** **Intervention group (*N* = 17)**		**Baseline—Final assessment** **Sham ultrasound group (*N* = 20)**		
*Secondary outcomes*		*Mean change (95% CI)*	*p value*	*Mean change (95% CI)*	*p value*	Interaction group^*^time
Cervical range of motion	*Movement*					*F; df; p value*
	Flexion	-2.20 (-8.71; 4.30)	0.496	-7.93 (-14.95; -0.91)	0.028^*^^CR^	2.72; 34; 0.108
	Extension	-5.14 (-13.64; 3.36)	0.227	-6.69 (-15.94; 2.55)	0.151	0.12; 34; 0.728
	Lateral flexion right	-8.12 (-13.31; -2.93)	0.003^*^^CR^	-5.84 (-11.48; -0.21)	0.042^*^^CR^	0.70; 34; 0.408
	Lateral flexion left	-5.61 (-12.13; 0.90)	0.089	-5.31 (-12.35; 1.71)	0.134	0.01; 34; 0.932
	Rotation right	-3.10 (-7.66; 1.46)	0.176^CR^	-0.36 (-5.33; 4.61)	0.883^CR^	1.34; 34; 0.255
	Rotation left	-5.43 (-10.30; -0.57)	0.030^*^^CR^	-7.01 (-12.36; -1.67)	0.012^*CR^	0.41; 34; 0.524
Pressure pain threshold	*Muscle*					
	Frontal	-0.37 (-0.77; 0.01)	0.058^CR^	0.04 (-0.39; 0.47)	0.860	4.30; 112; 0.040^*^
	Anterior temporalis	-0.46 (-0.80; -0.11)	0.009*^.CR^	-0.23 (-0.61; 0.14)	0.219	1.65; 112; 0.201
	Medium temporalis	-0.42 (-0.87; 0.03)	0.067^CR^	-0.06 (-0.55; 0.43)	0.808	2.43; 112; 0.122
	Posterior temporalis	-0.78 (-1.29; -0.27)	0.003*^CR^	-0.29 (-0.84; 0.26)	0.305	3.64; 112; 0.059
	Sternocleidomastoid	-0.14 (-0.54; 0.26)	0.488	0.06 (-0.38; 0.50)	0.791	0.94; 112; 0.334
	Scapular levator	-0.22 (-0.78; 0.33)	0.422^CR^	-0.10 (-0.71; 0.51)	0.749	0.20; 112; 0.657
	Upper trapezius	-0.26 (-0.42; -0.10)	0.002* ^CR^	-0.22 (-0.40; -0.04)	0.017^*^	0.28; 112; 0.595
	Suboccipital	-0.34 (-0.67; -0.02)	0.036* ^CR^	-0.18 (-0.54; 0.17)	0.305	0.95; 112; 0.331
CCFT	*Pressure stage*					
		-0.95 (-2.40; 0.49)	0.192	-3.15 (-4.53; -1.77)	0.001^*^	2.60; 70; 0.081
MIVC	*Muscle group*					
	Flexor					
	Force_n_ (N/kg)	0.04 (-0.13; 0.20)	0.639	0.14 (-0.05; 0.31)	0.142	1.23; 34; 0.275
	Peak time (s)	2.80 (-0.58; 6.19)	0.102^CR^	2.73 (-0.88; 6.35)	0.134^CR^	0.00; 34; 0.971
	Extensor					
	Force_n_ (N/kg)	-0.11 (-0.46; 0.24)	0.530^CR^	0.18 (-0.19; 0.57)	0.327	2.57; 34; 0.118
	Peak time (s)	-4.65 (-8.42; -0.89)	0.017^*^	-3.98 (-8.02; 0.05)	0.053	0.11; 34; 0.745
Endurance	*Muscle group*					
	Flexor					
	Endurance time (s)	-9.64 (-29.64; 10.34)	0.334	-9.70 (-31.57; 12.16)	0.373	0.00; 34; 0.995
	Extensor					
	Endurance time (s)	65.15 (-33.98; 164.29)	0.190	51.87 (-54.82; 158.58)	0.330	0.07; 33; 0.796
Questionnaires	*Total score*					
	MIDAS	13.64 (-11.03; 38.33)	0.269	3.16 (-23.40; 29.73)	0.810	0.62; 33; 0.436
	NDI	-2.22 (-6.47; 2.03)	0.292	-6.90 (-12.50; -1.29)	0.018^*^	3.90; 24; 0.059
	ASC-12	1.20 (-1.64; 4.05)	0.396	-0.12 (-3.21; 2.97)	0.938	0.79; 34; 0.380
	TAMPA	-0.10 (-4.67; 4.46)	0.963	-1.83 (-6.87; 3.21)	0.465	0.58; 34; 0.452
	PSFS	-1.90 (-2.96; -0.84)	0.001^*^	-1.77 (-2.93; -0.61)	0.003^*^	0.05; 173; 0.816

*95% CI* 95% confidence interval, *CCFT* craniocervical flexion test, *MIVC* maximal isometric voluntary contraction, *Force*_*n*_ force normalized by the subject mass, *s* seconds, *MIDAS* Migraine Disability Assessment *NDI* Neck Disability Index, *ASC-12* 12 item Allodynia Symptom Checklist, *TAMPA* Tampa Scale for Kinesiophobia, *PSFS* Patient-specific Functional Scale

^*^p < 0.05

^CR^clinically relevant

Pressure pain threshold: There was a significant improvement in the sensitivity of the frontal muscle (F = 4.30; df = 112; *p* = 0.040) in favor of the intervention group (Table 3). Within-group clinical relevance was observed for all muscles in the intervention group, except the SCM. No clinically relevant results were observed in the sham ultrasound group (Table 3 and Additional file 3).

Craniocervical flexion test: No significant differences were observed in the group-by-time interaction (F = 2.60; df = 70; *p* = 0.081) (Table 3). However, there was a significant reduction in the electromyographic activity of the upper trapezius muscle in the intervention group (*p* = 0.023) in the fourth stage (28 mmHg) of the test, and the anterior scalene muscle in the fourth (28 mmHg) (*p* = 0.030) and fifth (30 mmHg) (*p* = 0.010) stages of the CCFT. The graphical representation of these results can be viewed in the Additional file 4.

Maximum isometric voluntary contraction: No statistically significant differences were observed in the group-by-time interaction (*p* > 0.05) for clinical data on the MIVC (Table 3). A clinically relevant result was observed in the intervention group for normalized strength during the cervical extensor MIVC and, in both groups, for the peak time of flexors (Table 3 and Additional file 3). There were no differences between the groups over time (*p* > 0.05) in the electromyographic data during this test.

Cervical muscle endurance test: For clinical data, there was no significant difference in group-by-time interaction (*p* > 0.05) (Table 3) neither clinically relevant results (Additional file 3). The intervention group showed significantly increased median frequencies of the splenius capitis muscle during the endurance test of the cervical flexors (*p* = 0.014) and the anterior scalene during the endurance test of the flexors (*p* < 0.000) and cervical extensors (*p* = 0.045) when comparing the baseline and final assessments (Additional file 4).

Questionnaires: No significant results were observed in the group-by-time interaction (*p* > 0.05) for all applied questionnaires (Table 3). For the PSFS questionnaire, there was a significant within-group increase in the total score for both the intervention and sham ultrasound groups (*p* = 0.001 and *p* = 0.003, respectively), demonstrating an improvement in functionality throughout the treatment.

**Fig. 2:**
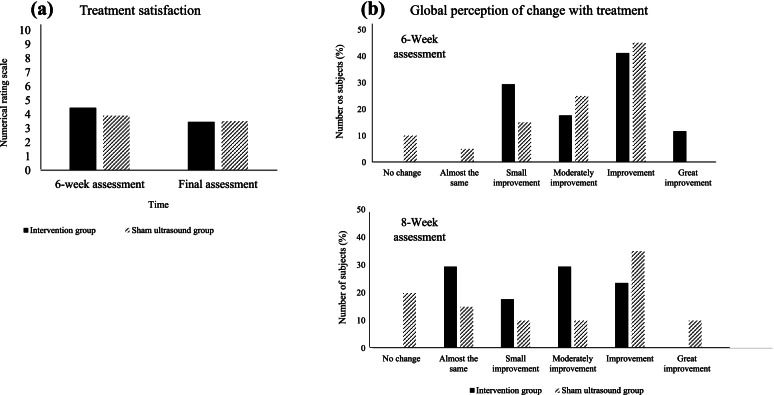
**(a)** mean of treatment satisfaction score, measured by the numerical rating scale (NRS), in both groups in the 6-week assessment and final assessment; where 0 is totally satisfied and 10 is totally dissatisfied **(b)** distribution of volunteers from both groups (IG = intervention group; SUG = sham ultrasound group) according to the individual's perception of change due to treatment, assessed during the 6-week assessment and final assessment (8-week assessment)

For the Global Perception of Change questionnaire, no significant group-by-time interaction (*p* > 0.05) was found. After six weeks of treatment, the mean of satisfaction in the intervention group was 4.47 (SD = 1.28), while that in the sham ultrasound group was 3.90 (SD = 1.33). At the end of eight weeks, the mean decreased in the intervention group to 3.47 (SD = 1.17) and in the sham ultrasound group to 3.50 (SD = 2.18) (Fig. 2A). The results also show that, for both groups, throughout the treatment, the improvement was categorized as “moderately improvement” to “improvement” (Fig. 2B).

## Discussion

Our hypothesis was not supported. The CMSE neither reduced the frequency and intensity of headache nor improved the performance and sensitivity of the cervical muscles, cervical range of motion, and migraine and neck pain-related disabilities. Although we did not observe repercussions in the physical examinations, the analysis of the electromyography revealed a better performance on the cervical muscles in the intervention group, observed by decreasing of RMS during the last stages of CCFT and the increase of median frequency during the endurance test.

Our baseline results demonstrated a between-groups difference for the presence and intensity of neck pain. Due to the important relationship between migraine and neck pain [9, 10], we considered the presence and intensity of neck pain as covariates in our analyzes to try to attenuate their influences on our results.

It has been established that aerobic exercise reduces the frequency of attacks in patients with migraine [15], which is considered one of the main non-pharmacological interventions used for chronic disease management [51]. Although we hypothesized that our patients would also benefit from CMSE due to musculoskeletal disorders and the pathophysiology of migraine and characteristics of nociplastic pain, our findings suggest that only the protocol proposed in this study is no better than sham ultrasound plus home-stretching for reducing the frequency and intensity of headache. However, based on the clinically relevant and electromyographic results, we suggest that in future studies this protocol should be considered again, increasing the treatment time or even associating it with other physical therapy techniques.

In clinical trials with manual therapy, placebo treatment is performed in different ways and the real placebo treatment does not exist. Therefore, sham therapies are used to verify the effectiveness of the applied technique [52]. However, in these clinical trials, sham treatment may have a greater effect on outcomes that depend on the patient’s report, such as pain [53–55], as in our study. We observed a reduction at headache intensity in both groups; both groups were satisfied regardless of the treatment performed. These results might be related to the fact that all volunteers felt treated and have been satisfied by inserted in a tertiary referral service.

The placebo effect cannot be ignored. Patients who received sham therapy were not only as satisfied with the treatment as patients in the intervention group, but also, had the greatest concentration in “improvement” at the end of treatment. In both groups, our results demonstrated that the group that performed the craniocervical exercises was not superior in the variables evaluated than the sham ultrasound group. Therefore, the protocol of CMSE was unable to overcome the improvements resulting from the placebo effect.

Another aspect to be observed was the performance of cervical stretching, performed by the sham ultrasound group. The improvement observed by the sham ultrasound group for the intensity of headache, as well as for cervical ROM gain and a better performance in CCFT may have been observed due to cervical stretching. Once the literature demonstrates that performing scapula-cervical stretching reduces neck pain and improves flexibility and muscle function, including in patients with migraine [56–58]. In addition, the level of physical activity of the volunteers in both groups was not evaluated, which could influence our results. However, these results should be analyzed with caution, since both peripheral strategies (stretching) and central strategies (exercises) were not significantly different.

Despite the non-significant effect on physical tests performance, the CMSE promoted a change in the activation of cervical muscles reducing the RMS during the CCFT and increasing the median frequency during endurance test. It corroborates the significant decrease in the cervical muscles RMS of patients with neck pain after performing craniocervical exercises [59]. It allows us to suggest that craniocervical exercise improves the activation of the cervical muscles in patients with migraine, but does not improve test performance.

We assume that the study has some limitations. The first is in relation to statistical analysis and the power of results. In an intention-to-treat (ITT) analysis, all randomized patients are included in the analysis in their assigned groups and an optimal ITT analysis occurs when a complete data set exists [60]. In cases of dropouts, lost participants can be disregarded or data imputed. Even in the face of this limitation, as the dropout number was below (11.9%) that stipulated in the literature (15%) [18], it was decided to exclude these patients from the analysis. We recognize that for primary outcomes the sample size calculation was based on previous studies and the expected differences of 3.3 for headache frequency and 1.5 for intensity were not achieved. Therefore, we suggest to adequate, in future studies, the sample size estimation considering the smaller effect size observed here so that the results are not underestimated.

Second, the use of only one technique (CMSE). However, it was necessary to verify the effectiveness of protocol alone. Other limitations include the impossibility of being a double-blind study, and the exercises performed at home were not monitored. Still, this study presents a low risk of bias [22], is the first to verify the effectiveness of CMSE in patients with migraine and highlights the importance of further research.

## Conclusion

Performing CMSE is not enough to reduce the frequency and intensity of headache or improve the performance of the cervical muscles and reduce migraine and neck pain-related disabilities.

## Supplementary Information

**Additional file 1.** Guidelinebook for sham ultrasound group.

**Additional file 2.** Clinical relevance of primary outcomes.

**Additional file 3.** Clinical relevance of secondary outcomes.

**Additional file 4:** **(a)** Representation of the mean of the normalized rootmean square (RMS) for the muscles anterior scalene and upper trapezius duringthe fourth (28 mmHg) and the fifth (30 mmHg) stage of the CCFT in the threeassessments for booth groups; *=*p*<0.05 for group-by-time interaction; **(b)** Representationof the mean of median frequency for muscles anterior scalene and spleniuscapitis during the flexor and extensor endurance test, in the two assessments, baselineand final, for booth groups; *=*p* < 0.05 for group-by-time interaction.

## Data Availability

The datasets generated and/or analysed during the current study are not publicly available due to participant confidentiality, but are available from the corresponding author on reasonable request.

## Abbreviations

ASC-12: 12-Item Allodynia Symptom Checklist
CCFT: Craniocervical Flexion Test
CMSE: Craniocervical Muscle-Strengthening Exercise
CROM: Cervical Range of Motion
ES: Effect Size
MID: Minimum Important Difference
MIDAS: Migraine Disability Assessment
MIVC: Maximum Isometric Voluntary Contraction
NDI: Neck Disability Index
NRS: Numerical Rating Scale
PSFS: Patient-Specific Functional Scale
RMS: Root Mean Square
SCM: Sternocleidomastoid
SD: Standard Deviation
TAMPA: Tampa Scale for Kinesiophobia
