# Supplementary material for: Neck-specific strengthening exercise compared with placebo sham ultrasound in patients with migraine: a randomized controlled trial
Source: BMC Neurol. 2022 Apr 2;22:126. doi: 10.1186/s12883-022-02650-0 (PMC8976325; doi:10.1186/s12883-022-02650-0)
Supplement: Supplementary file 1 — Additional file 1. Guidelinebook for sham ultrasound group. [file 12883_2022_2650_MOESM1_ESM.docx]

**
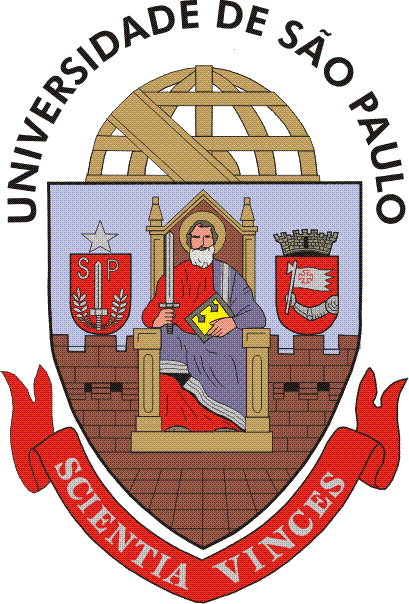
Additional file 1:** Guideline book for sham ultrasound group.


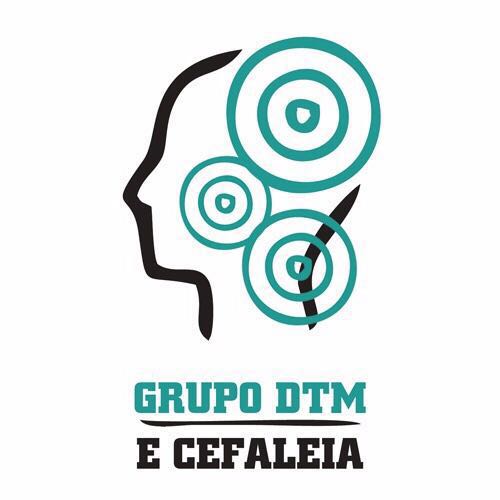

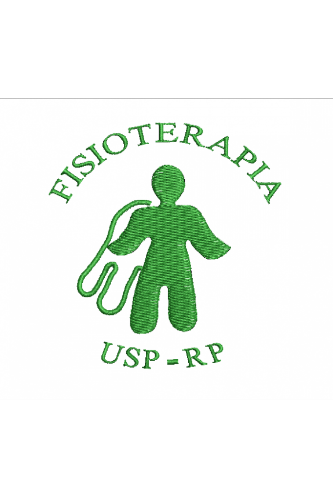

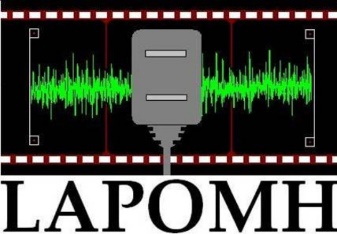


**Cartilha de Orientações e Exercícios**

**Ambulatório de DTM e Cefaleia**

Cartilha da (o): __________________________________________________________

**Como é a Enxaqueca?**


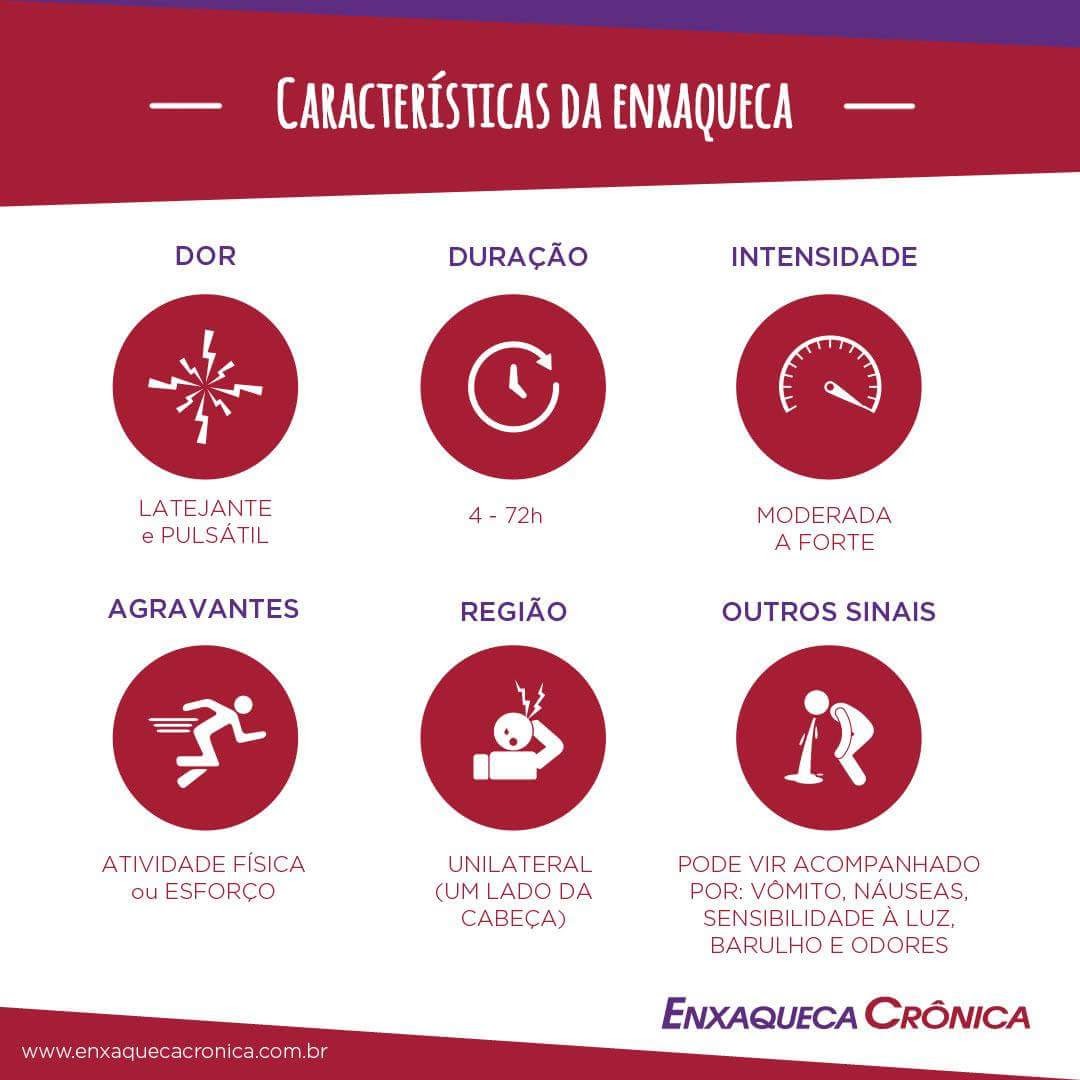


www.enxaquecacronica.com.br

**O que pode desencadear minha crise?**


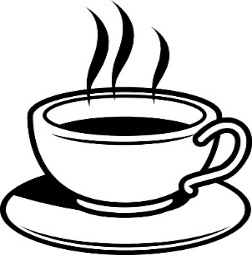

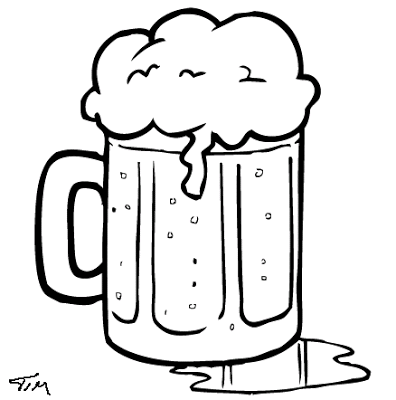

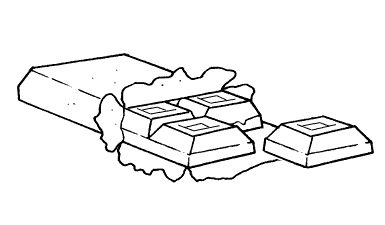


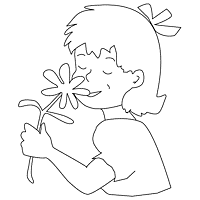


- Alguns alimentos, como o café, bebidas alcoólicas, chocolate, queijos, embutidos
- Odores (bons ou ruins), sons e luzes fortes
- Muitas ou poucas horas de sono
- Estresse, ansiedade, sedentarismo
- Período menstrual
- Abuso de remédios


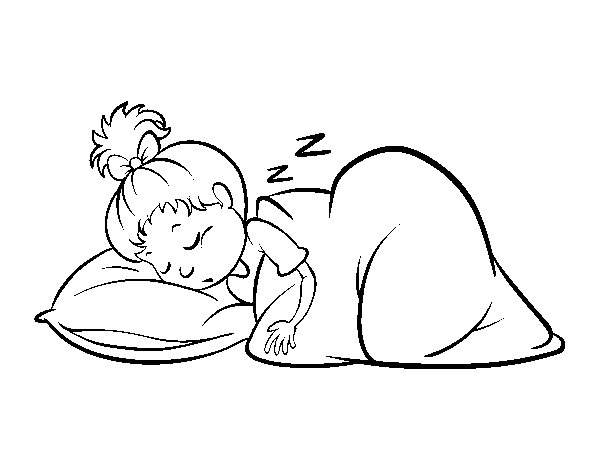

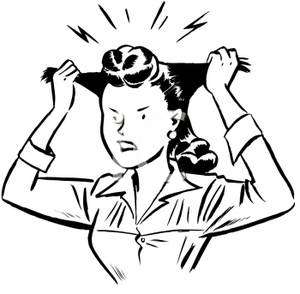


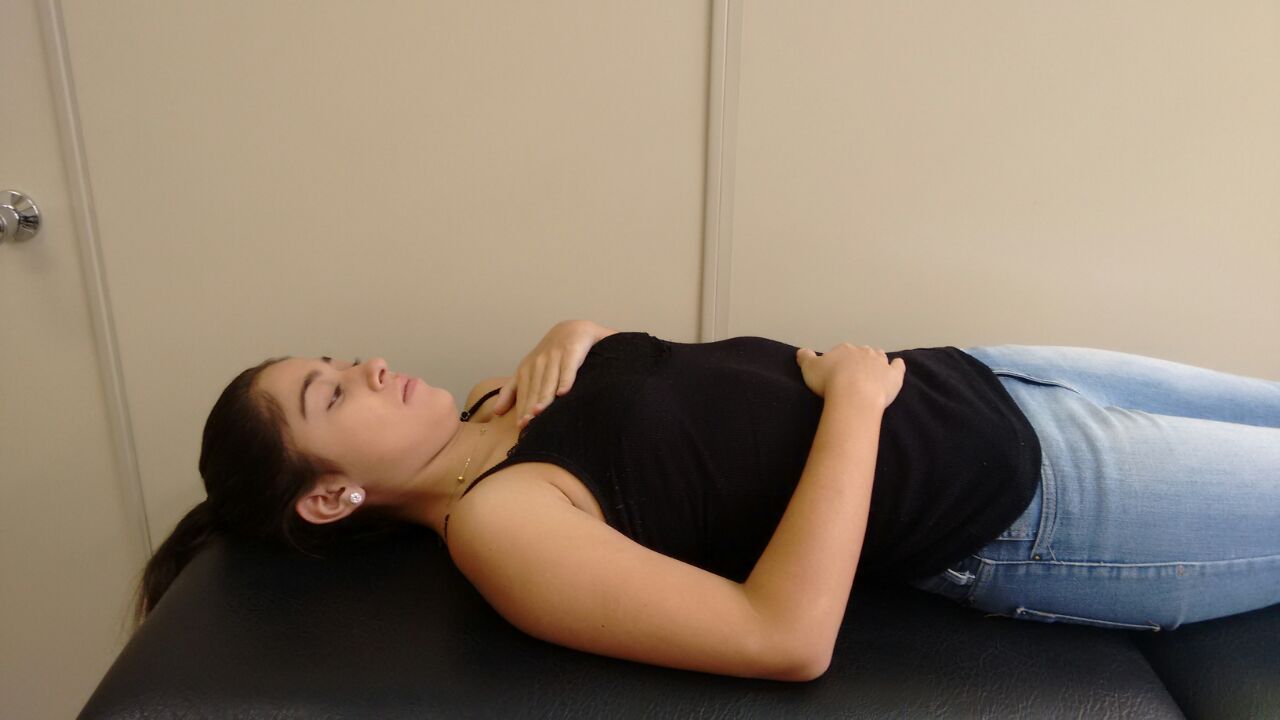
**Respiração**


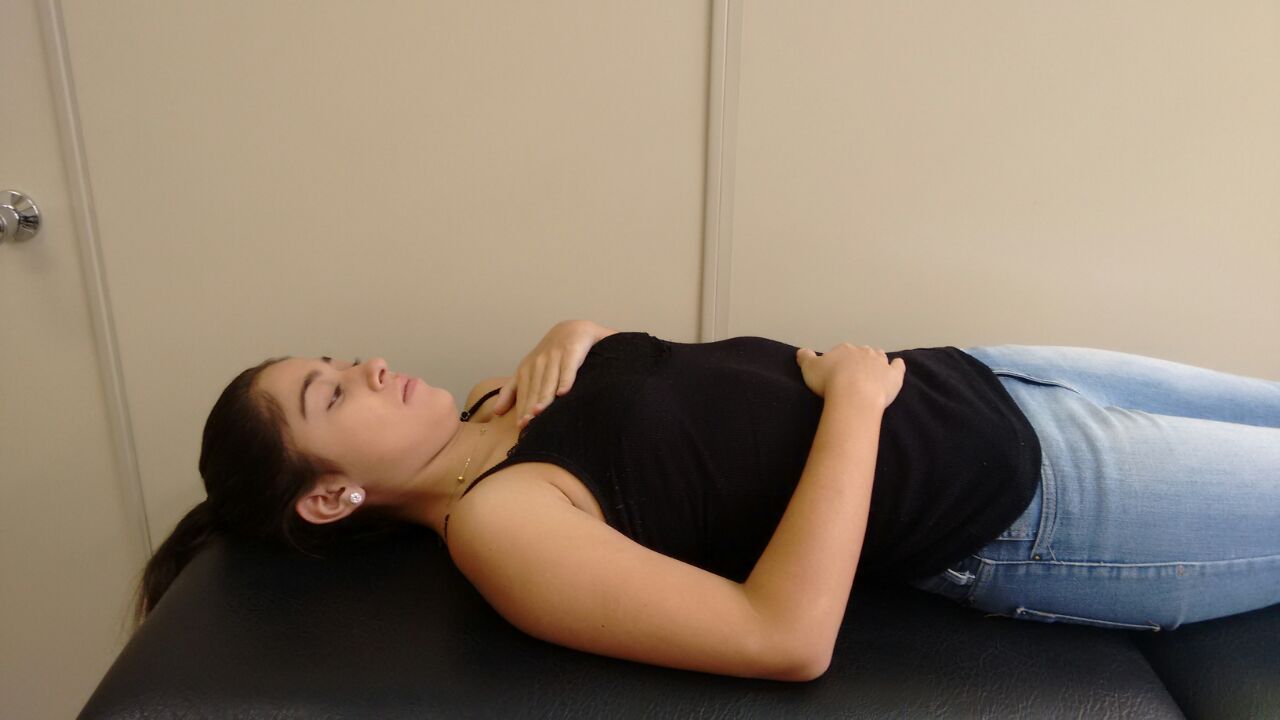


**Expiração:** Solte o ar lentamente pela boca esvaziando a barriga e descendo o peito.

**Inspiração:** Puxe o ar pelo nariz lentamente enchendo a barriga.

Para que você alcance os resultados desejados da melhor forma possível e em menor tempo é muito importante que **VOCÊ** participe **ativamente** do seu tratamento. Para isso criamos essa cartilha que contém alongamentos e exercícios orientados pelo (a) seu/sua fisioterapeuta para que você realize diariamente, em casa.

**
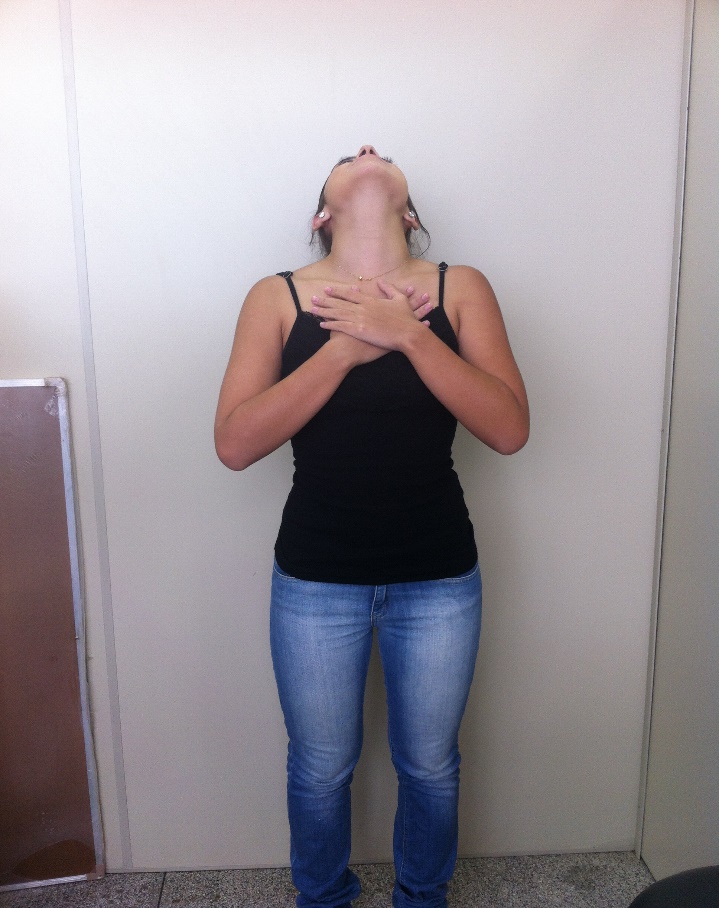

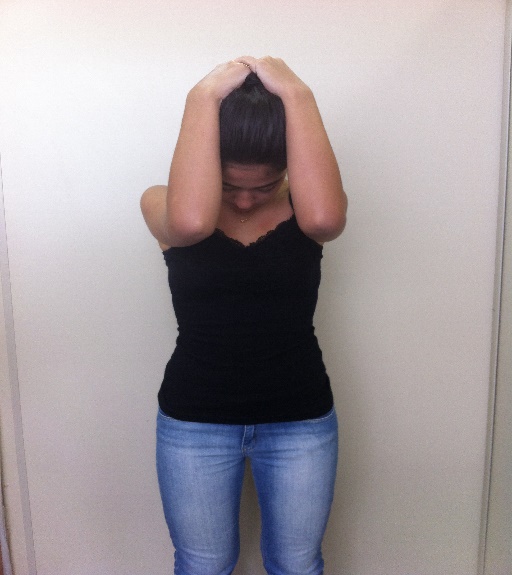
Alongamentos**

Coloque as mãos sobre o peito puxando para baixo e leve a cabeça para trás.

**Mantenha a posição e conte até 30.**

___ X ao dia

Coloque as duas mãos atrás da cabeça puxe para frente, em direção ao peito.

**Mantenha a posição e conte até 30.**

____ X ao dia


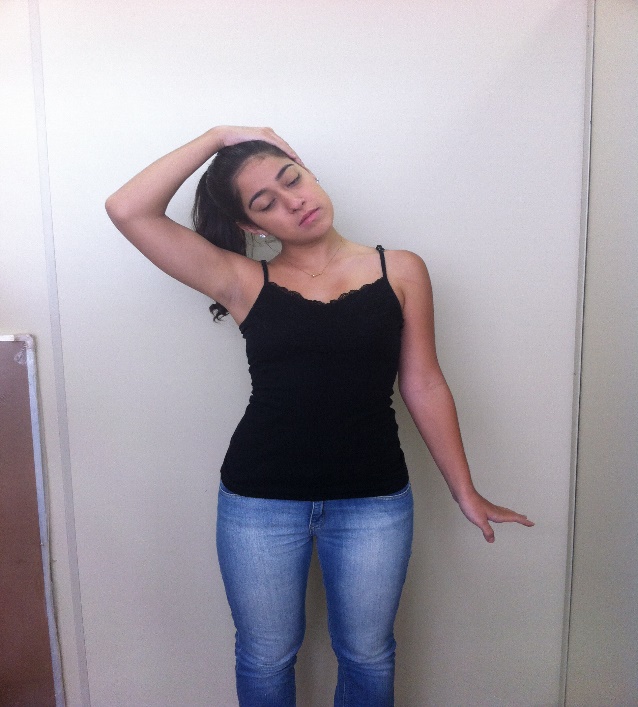

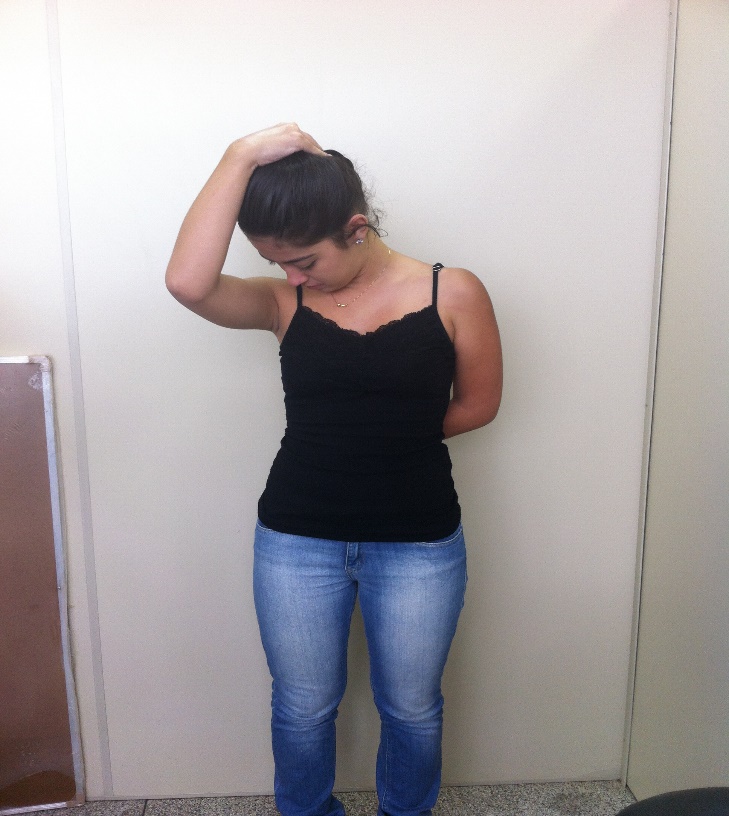


Coloque a mão sobre a orelha e puxe a cabeça em direção ao ombro, mantenha o olhar para frente e relaxe o ombro.

**Mantenha a posição e conte até 30.**

Incline a cabeça como se fosse olhar abaixo do braço e com a mão puxe a cabeça na mesma direção.

**Mantenha a posição e conte até 30.**

**Fazer para a direita e para a esquerda!**


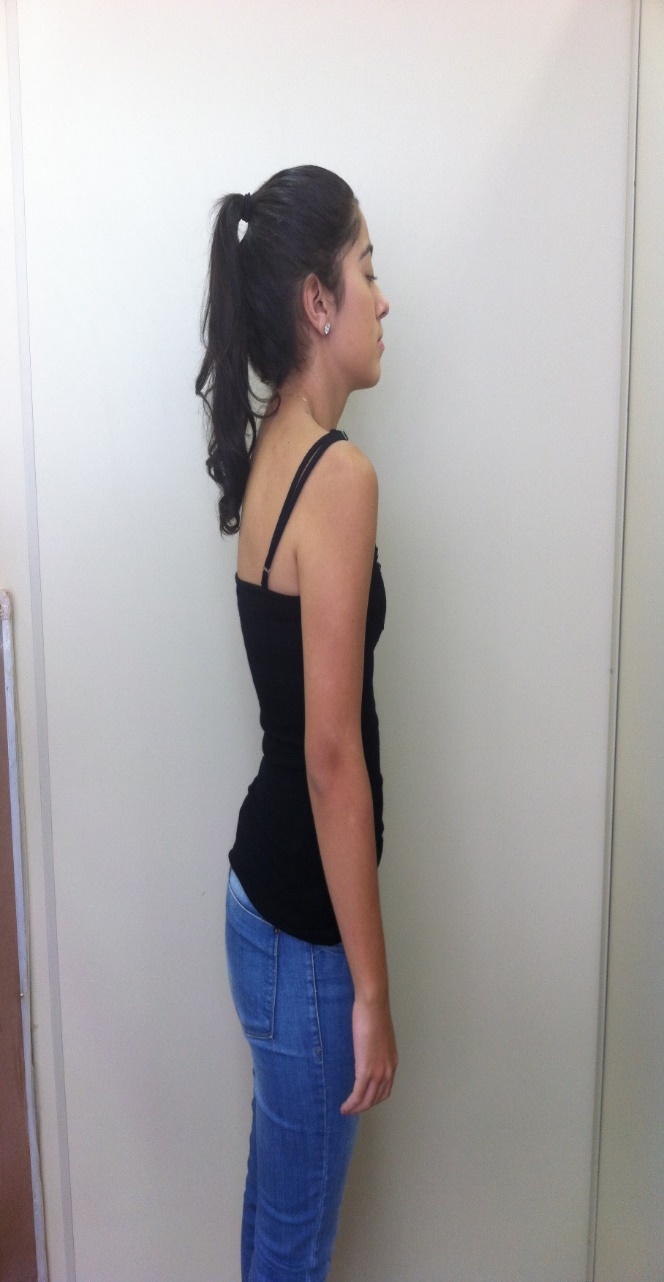

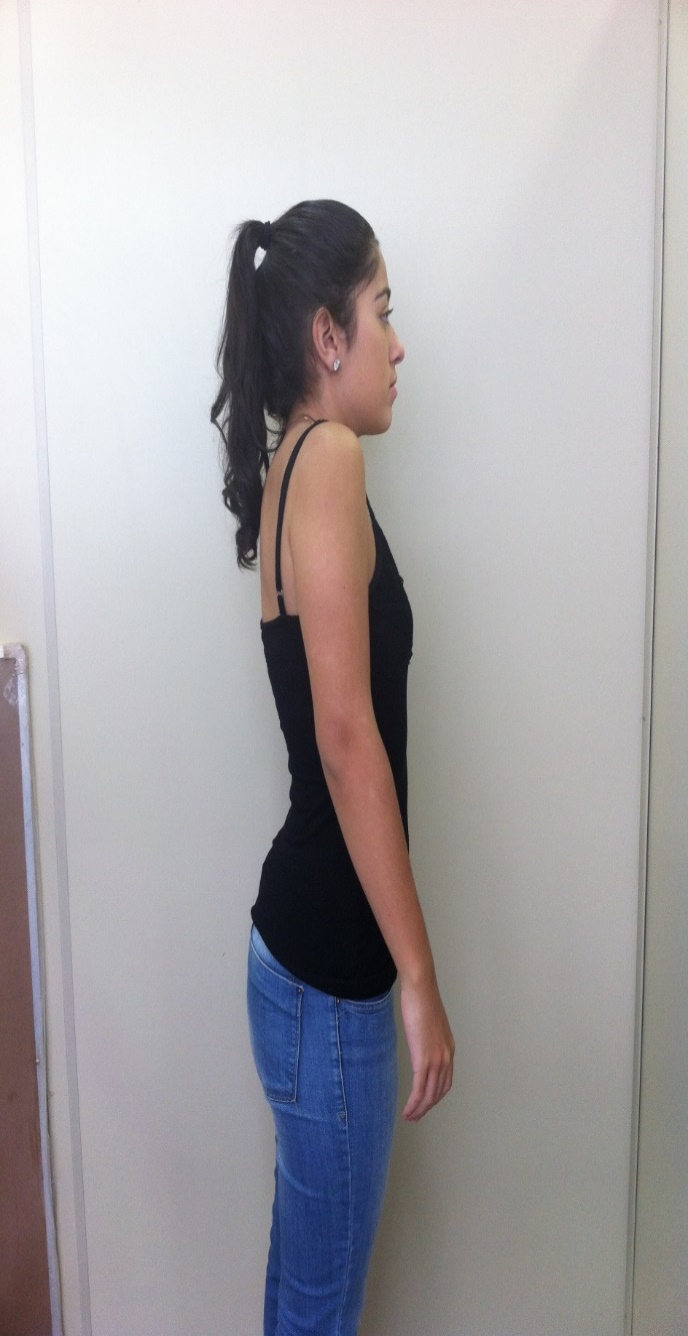

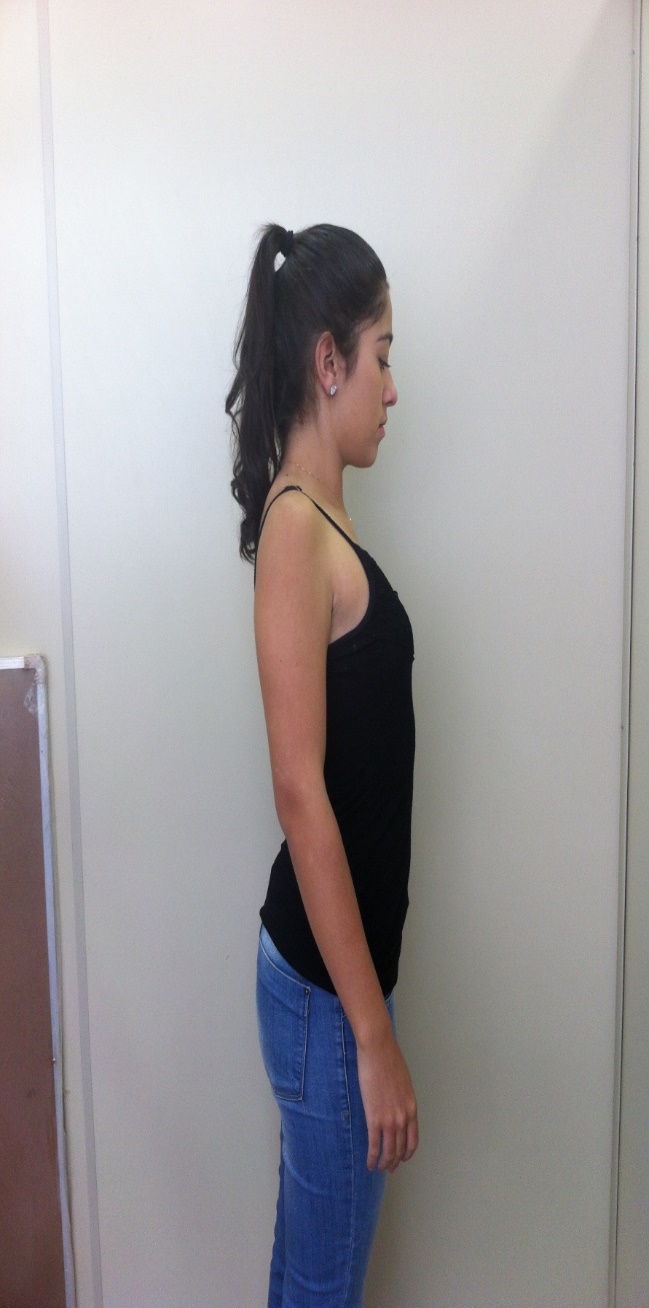

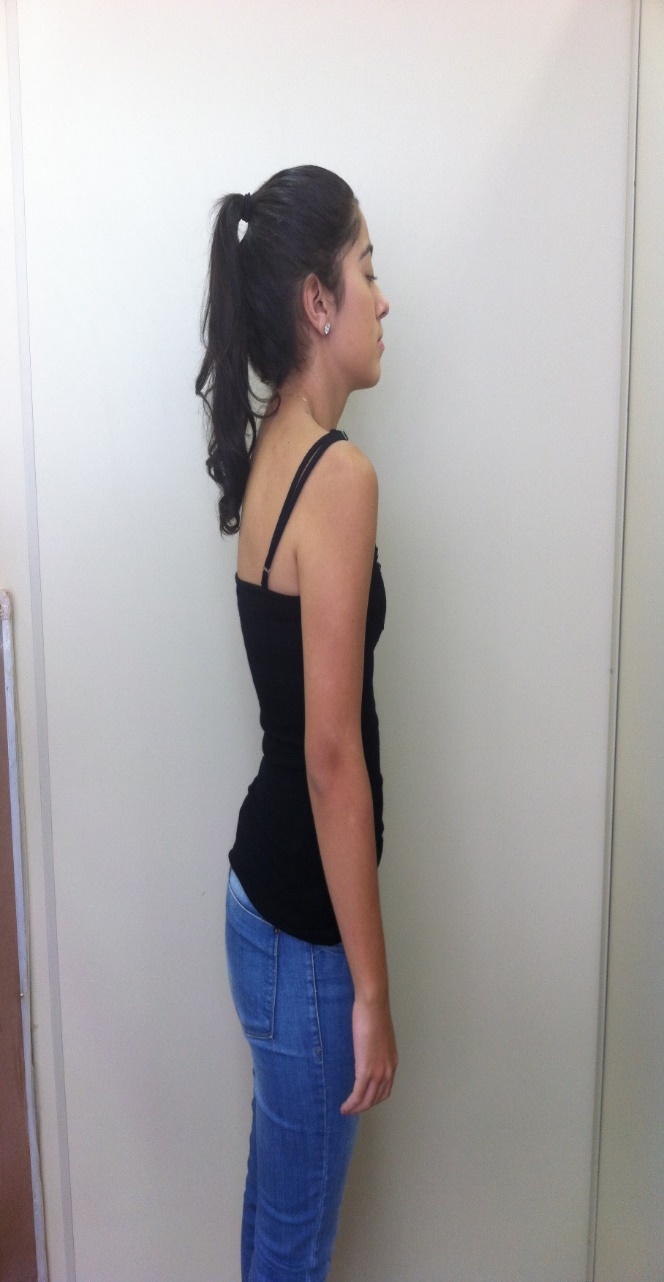

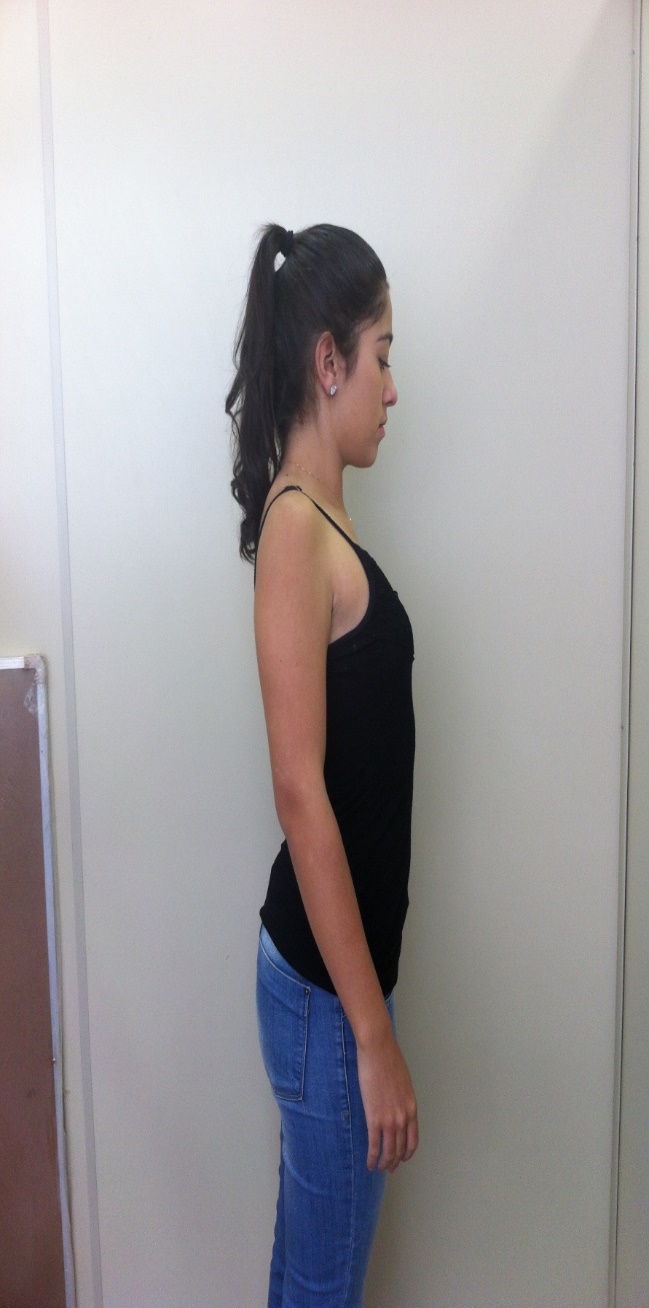


Em pé, faça círculos com os seus ombros, levando-os para a frente, para cima e para trás.

Se você quiser, pode fazer na frente de um espelho.

Faça 10 vezes

_____ X ao dia

Dicas e Observações
