# Supplementary material for: Neck-specific strengthening exercise compared with placebo sham ultrasound in patients with migraine: a randomized controlled trial
Source: BMC Neurol. 2022 Apr 2;22:126. doi: 10.1186/s12883-022-02650-0 (PMC8976325; doi:10.1186/s12883-022-02650-0)
Supplement: Supplementary file 2 — Additional file 2. Clinical relevance of primary outcomes. [file 12883_2022_2650_MOESM2_ESM.docx]

**Additional file 2:** Clinical relevance of primary outcomes.

|  |  | Baseline  Final assessment | | | | Baseline  Follow-up 1 | | | | Baseline  Follow-up 2 | | | | Baseline  Follow-up 3 | | | | |
| --- | --- | --- | --- | --- | --- | --- | --- | --- | --- | --- | --- | --- | --- | --- | --- | --- | --- | --- |
|  |  | *Mean change* | *Effect size* | *MID* | *Class.* | *Mean change* | *Effect size* | *MID* | *Class.* | *Mean change* | *Effect size* | *MID* | *Class.* | *Mean change* | *Effect size* | *MID* | *Class.* |  |
| *Primary outcomes* | *Groups* |  |  |  |  |  |  |  |  |  |  |  |  |  |  |  |  |  |
| Frequency of headache^¥^ | IG  SUG | 0.75  1.42 | 0.09  0.22 | 3.97  3.26 | NCR  NCR | 0.81  1.27 | 0.10  0.18 | 4.02  3.58 | NCR  NCR | 1.69  0.67 | 0.21  0.09 | 3.99  3.59 | NCR NCR | 2.05  0.27 | 0.31  0.04 | 3.28  3.60 | NCR  NCR |  |
| Intensity of pain (NRS) | IG  SUG | 3.02  3.76 | 2.22  2.39 | 0.68  0.79 | **CR**  **CR** | 3.38  2.81 | 2.07  1.52 | 0.82  0.93 | **CR**  **CR** | 4.02  3.56 | 2.48  2.02 | 0.81  0.88 | **CR**  **CR** | 4.08  3.41 | 2.80  1.95 | 0.73  0.87 | **CR**  **CR** |  |

MID=minimum important difference; Class=classification; ^¥^=represented in days with headache per month; IG=intervention group; SUG=sham ultrasound group; NRS=numerical rating scale; NCR=no clinically relevant; CR=clinically relevant.
