# Supplementary material for: Neck-specific strengthening exercise compared with placebo sham ultrasound in patients with migraine: a randomized controlled trial
Source: BMC Neurol. 2022 Apr 2;22:126. doi: 10.1186/s12883-022-02650-0 (PMC8976325; doi:10.1186/s12883-022-02650-0)
Supplement: Supplementary file 3 — Additional file 3. Clinical relevance of secondary outcomes. [file 12883_2022_2650_MOESM3_ESM.docx]

**Additional file 3:** Clinical relevance of secondary outcomes.

|  |  |  | Baseline  Final assessment | | | |
| --- | --- | --- | --- | --- | --- | --- |
|  |  |  | *Mean change* | *Effect size* | *MID* | *Classification* |
| *Secondary outcomes* |  |  |  |  |  |  |
| Cervical range of motion | *Movement* | *Groups* |  |  |  |  |
|  | Flexion | IG  SUG | 0.30  -4.41 | 0.03  -0.56 | 4.85  3.97 | NCR  **CR** |
|  | Extension | IG  SUG | -4.32  -3.93 | -0.33  -.033 | 6.48  5.95 | NCR  NCR |
|  | Lateral flexion right | IG  SUG | -6.42  -3.40 | -0.85  -0.46 | 3.77  3.68 | **CR**  **CR** |
|  | Lateral flexion left | IG  SUG | -3.49  -2.23 | -0.38  -0.26 | 4.55  4.25 | NCR  NCR |
|  | Rotation right | IG  SUG | -5.57  -2.86 | -0.71  -0.51 | 3.95  2.81 | **CR**  **CR** |
|  | Rotation left | IG  SUG | -3.71  -3.93 | -0.42  -0.50 | 4.47  3.91 | **CR**  **CR** |
| Pressure pain threshold | *Muscles* | *Groups* |  |  |  |  |
|  | Frontal | IG  SUG | -0.44  0.00 | -0.60  0.00 | 0.37  0.50 | **CR**  NCR |
|  | Anterior temporalis | IG  SUG | -0.39  -0.15 | -0.54  -0.17 | 0.36  0.45 | **CR**  NCR |
|  | Medium temporalis | IG  SUG | -0.49  -0.10 | -0.50  -0.10 | 0.49  0.50 | **CR**  NCR |
|  | Posterior temporalis | IG  SUG | -0.78  -0.26 | -0.72  -0.23 | 0.54  0.57 | **CR**  NCR |
|  | Sternocleidomastoid | IG  SUG | -0.34  -0.17 | -0.39  -0.30 | 0.44  0.29 | NCR  NCR |
|  | Scapular levator | IG  SUG | -0.51  -0.39 | -0.45  -0.29 | 0.56  0.67 | **CR**  NCR |
|  | Upper trapezius | IG  SUG | -0.50  -0.22 | -0.51  -0.21 | 0.49  0.52 | **CR**  NCR |
|  | Suboccipital | IG  SUG | -0.34  -0.14 | -0.71  -0.26 | 0.24  0.27 | **CR**  NCR |
| MIVC | *Muscle group* |  |  |  |  |  |
|  | Flexor |  |  |  |  |  |
|  | Force_n_ (N/kg) | IG  SUG | -0.09  -0.02 | -0.29  -0.11 | 0.16  0.09 | NCR  NCR |
|  | Peak time (s) | IG  SUG | 0.25  0.28 | 0.57  0.59 | 0.22  0.24 | **CR**  **CR** |
|  | Extensor |  |  |  |  |  |
|  | Force_n_ (N/kg) | IG  SUG | -0.24  0.04 | -0.44  0.09 | 0.28  0.24 | **CR**  NCR |
|  | Peak time (s) | IG  SUG | -0.10  -0.05 | -0.19  -0.13 | 0.27  0.20 | NCR  NCR |
| Endurance | *Muscle group* |  |  |  |  |  |
|  | Flexor |  |  |  |  |  |
|  | Endurance time (s) | IG  SUG | -6.05  -5.30 | -0.17  -0.19 | 17.54  14.03 | NCR NCR |
|  | Extensor |  |  |  |  |  |
|  | Endurance time (s) | IG  SUG | 24.05  3.89 | 0.17  0.02 | 71.31  79.94 | NCR NCR |

MID= minimum important difference; IG=intervention group; SUG=sham ultrasound group; NCR=no clinically relevant; CR=clinically relevant; MIVC=maximal isometric voluntary contraction; Force_n_=force normalized by the subject mass; s=seconds.
