# Supplementary material for: Neck-specific strengthening exercise compared with placebo sham ultrasound in patients with migraine: a randomized controlled trial
Source: BMC Neurol. 2022 Apr 2;22:126. doi: 10.1186/s12883-022-02650-0 (PMC8976325; doi:10.1186/s12883-022-02650-0)
Supplement: Supplementary file 4 — Additional file 4: (a) Representation of the mean of the normalized rootmean square (RMS) for the muscles anterior scalene and upper trapezius duringthe fourth (28 mmHg) and the fifth (30 mmHg) stage of the CCFT in the threeassessments for booth groups; *=p<0.05 for group-by-time interaction; (b) Representationof the mean of median frequency for muscles anterior scalene and spleniuscapitis during the flexor and extensor endurance test, in the two assessments, baselineand final, for booth groups; *=p < 0.05 for group-by-time interaction. [file 12883_2022_2650_MOESM4_ESM.docx]

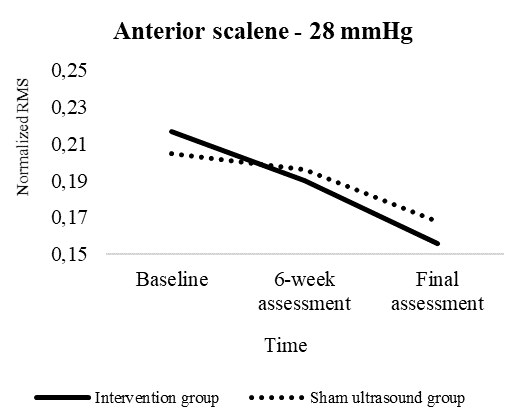


***p=0.030****


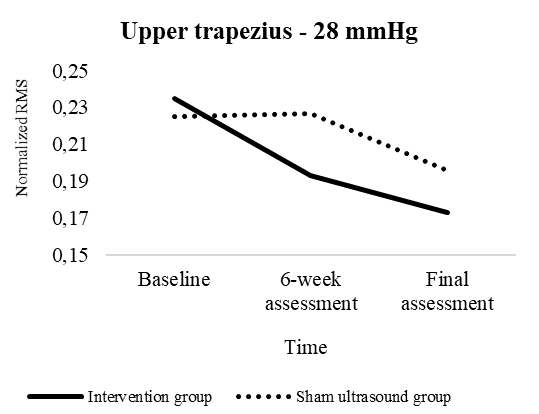


***p=0.028****


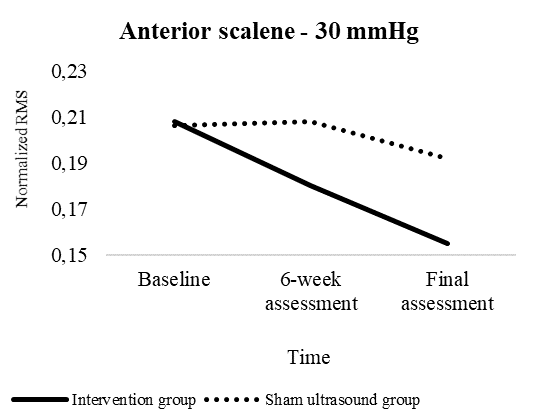


***p=0.010****

**(a)**


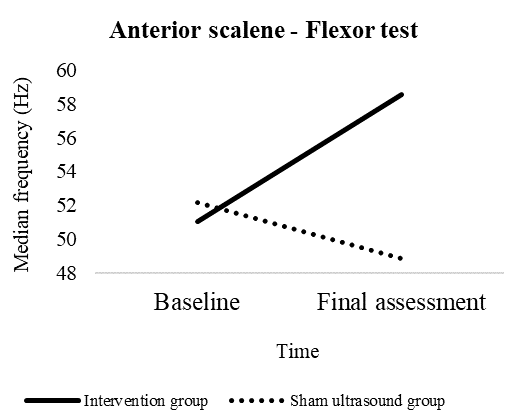


***p<0.000****


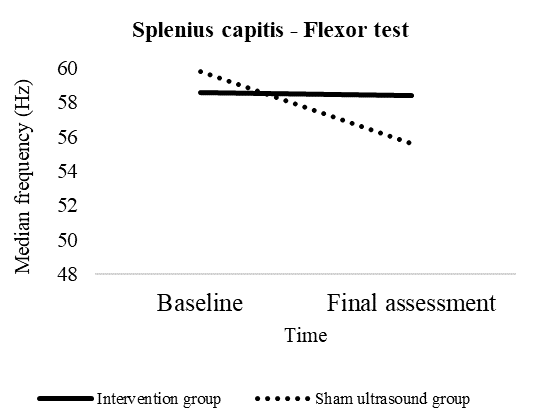


***p=0.014****


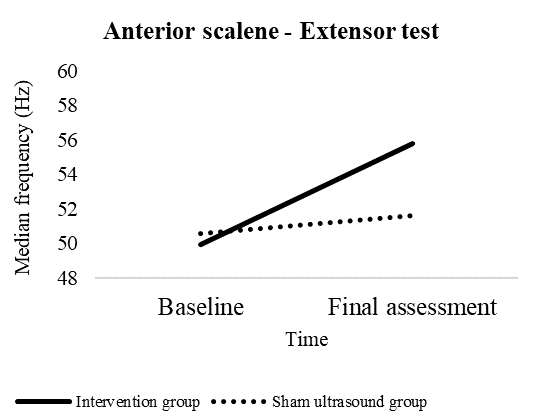


***p=0.045****

**(b)**

p=0,926

p=0,063

p=0,023

Interação: p=0,010

**Additional file 4. (a)** Representation of the mean of the normalized root mean square (RMS) for the muscles anterior scalene and upper trapezius during the fourth (28 mmHg) and the fifth (30 mmHg) stage of the CCFT in the three assessments for booth groups; *=p<0.05 for group-by-time interaction; **(b)** Representation of the mean of median frequency for muscles anterior scalene and splenius capitis during the flexor and extensor endurance test, in the two assessments, baseline and final, for booth groups; *=p<0.05 for group-by-time interaction.
